# Limitations of the rhesus macaque draft genome assembly and annotation

**DOI:** 10.1186/1471-2164-13-206

**Published:** 2012-05-30

**Authors:** Xiongfei Zhang, Joel Goodsell, Robert B Norgren,

**Affiliations:** 1 Department of Genetics, Cell Biology and Anatomy, University of Nebraska Medical Center, Omaha, NE, 68198, USA

## Abstract

Finished genome sequences and assemblies are available for only a few vertebrates. Thus, investigators studying many species must rely on draft genomes. Using the rhesus macaque as an example, we document the effects of sequencing errors, gaps in sequence and misassemblies on one automated gene model pipeline, Gnomon. The combination of draft genome with automated gene finding software can result in spurious sequences. We estimate that approximately 50% of the rhesus gene models are missing, incomplete or incorrect. The problems identified in this work likely apply to all draft vertebrate genomes annotated with any automated gene model pipeline and thus represent a pervasive challenge to the analysis of draft genomes.

## Background

Genomic sequences and assemblies have been provided for many vertebrate species. However, only a few have reached "finished" status. The rest are considered "draft" genomes. Although draft genomes are known to be less complete than finished genomes, the implications of working with resources derived from draft genomes is not always appreciated by investigators not involved in their production. Independent assessments of possible misassemblies and sequencing errors are rarely done. Finally, the quality metrics reported when a new genome is published provide a global assessment of completeness but provide little information on the quality of the gene models derived from the sequence and assemblies.

To address these issues, we examined the draft genome sequence and assembly of the rhesus macaque (*Macaca mulatta*) [1], an important biomedical research model [2-8]. Like many vertebrate draft genomes, an animal was sequenced to 5-6-fold coverage using Sanger sequencing. Several different assemblers were used and their results combined [1]. The human genome assembly was used to correct errors in the rhesus assembly.

Using a gene-based approach that incorporated both similarity and synteny information from the human genome, we found sequencing errors, missing sequence which included exons or parts of exons, and misassemblies in the draft rhesus genome. Further, the automated annotation pipeline used by NCBI, Gnomon [9], made a wide variety of errors in producing gene models. These errors were partially due to incorrect sequence and assembly information and partly due to the spurious annotations Gnomon created when presented with incomplete data.

We provide specific examples of the types of errors produced in the draft genome and in the annotation provided by Gnomon. We estimate that approximately 50% of the rhesus macaque genes were misannotated as a result. The errors observed in the current work likely apply to most other draft vertebrate genomes and would be expected to occur with any automated gene model pipeline.

## Results

### Sequencing errors

#### Case 1

actin-related protein T1 (ACTRT1):

incorrect insertion results in a frameshift

**Figure 1:**
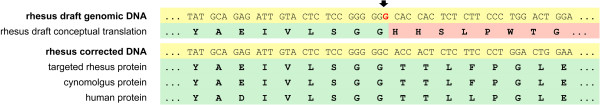
**ACTRT1. Incorrect insertion results in a frameshift.** Arrow points to a sequencing error (incorrect insertion) in the rhesus draft sequence for the single ACTRT1 exon. The reverse complement is shown to facilitate comparison with the corrected sequence and translated proteins. Red "G" indicates sequencing error. Yellow highlighting indicates nucleotide sequence. Green highlighting indicates correct protein sequence. Pink highlighting indicates spurious protein sequence caused by the sequencing error (insertion)

Our targeted sequencing of the single exon of the rhesus ACTR1 gene [GenBank:JF749838.1] revealed that a "C" was incorrectly inserted at position 126,268,430 in GenBank:NC_007878.1 (Figure 1). This occurred within the coding region of the single exon of this gene and thus resulted in a frameshift. The GenBank RNA associated with this gene was altered to remove the frameshift, but it is still annotated as a pseudogene [GenBank:XR_010864.2]. No protein associated with this gene has been derived from the draft rhesus genome sequence by NCBI. ACTR1 RNA sequences [GenBank:AB168640.1, BAE00751.1] from a monkey species closely related to rhesus macaques - cynomolgus macaques (*Macaca fascicularis*) - translate to a protein sequence [GenBank:Q4R821.1] which is a good match for the rhesus protein sequence without the frameshift.

#### Case 2

adrenergic, beta-1-, receptor (ADRB1):

incorrect sequence results in a premature stop codon

**Figure 2:**
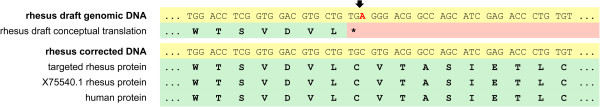
**ADBR1. Incorrect sequence results in a premature stop codon.** Arrow points to a sequencing error ("A" instead of "C") in the rhesus draft sequence for the single ADBR1 exon. Yellow highlighting indicates nucleotide sequence. Green highlighting indicates correct protein sequence. Pink highlighting indicates premature truncation of protein sequence caused by the sequencing error

The rhesus locus syntenic to the ADRB1 gene[GenBank:NC_007866.1; positions 113,704,808 - 113,707,697] is annotated as a pseudogene by NCBI in the Gene database [10]http://www.ncbi.nlm.nih.gov/gene/100426598. This is likely because attempts to translate the single exon at this locus results in a stop codon. However, our targeted sequencing in this region [GenBank:JN589014.1]) reveals that sequencing errors have led to the apparent stop codon (Figure 2). Replacing positions 113,705,260 - 113,705,609 with our sequence [GenBank:JN589014.1] results in a protein sequence which is very similar to the human ortholog [GenBank:NP_000675.1] (96.6% identical, one gap). Another rhesus macaque genomic sequence [GenBank:X75540.1] submitted by another group yields the same protein sequence as our sequence [GenBank:JN589014.1]. Thus, it seems likely the reference genome sequence is incorrect. This sequencing error resulted in Gnomon incorrectly marking the rhesus ADBR1 locus a pseudogene.

#### Case 3

adenylate cyclase 3 (ADCY3):

missing exon results in spurious sequence

**Figure 3:**
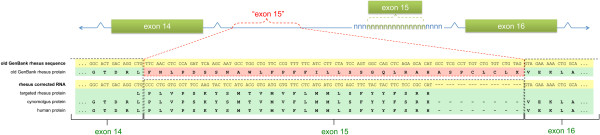
**ADCY3. Missing exon results in spurious sequence.** Red "exon 15" indicates false exon created by Gnomon from intronic sequence. Correct exons are indicated in green boxes. Green letters at the bottom of the panel indicate boundaries of correct exons. Yellow highlighting indicates nucleotide sequence. Green highlighting indicates correct protein sequence. Pink highlighting indicates spurious protein sequence caused by the false exon

The putative mRNA sequence identified by Gnomon from the draft rhesus genome [GenBank:XM_002799164.1] for the rhesus ADCY3 gene translated to a protein for which a span of amino acids were a poor match for the human ortholog (Figure 3). In part, this was because Gnomon selected intronic sequence to create a spurious exon 15 when it could not find the real sequence. Our targeted sequencing of exon 15 of the rhesus ADCY3 gene [GenBank:HM067826.1] revealed that this sequence was missing from the rhesus draft assembly. A gap at position 24,741,526 - 24,742,078 of [GenBank:NC_ 0078870.1] likely includes the region where this sequence should have appeared. The subsequence of [GenBank:HM067826.1] which spans exon 15, 516–597, matches 100% with a cynomolgus macaque cDNA clone [GenBank:AB172471.1]. The NCBI mRNA for this gene now incorporates the sequence we identified for exon 15 [GenBank:NM_001205221.1].

#### Case 4

aminoadipate aminotransferase (AADAT):

missing exon results in spurious protein sequence

**Figure 4:**
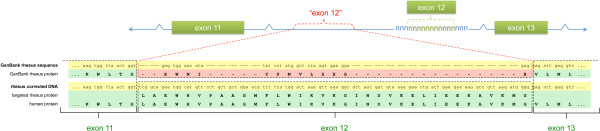
**AADAT. Missing exon results in spurious sequence.** Red "exon 12" indicates false exon created by Gnomon from intronic sequence. Correct exons are indicated in green boxes. Green letters at the bottom of the panel indicate boundaries of correct exons. Yellow highlighting indicates nucleotide sequence. Green highlighting indicates correct protein sequence. Pink highlighting indicates spurious protein sequence caused by the false exon

The GenBank protein sequence for the rhesus ortholog of the AADAT gene [GenBank:XP_002804303.1] contains approximately 180 amino acids not found in other species. However, this striking difference is most likely due to misannotation rather than evolutionary divergence. Alignment of the human AADAT transcript [GenBank:NM_182662.1] with the rhesus draft genome reveals that exon 12 is missing in the rhesus sequence (Figure 4). We targeted this exon for sequencing in the rhesus [GenBank:JN624744.1]. When our exon 12 sequence is used to create a gene model for rhesus AADAT, a protein similar to the human ortholog is found in this region. However, we were not able to create a complete protein from the available sequence because another 30 nucleotides of sequence in exon 13 were also missing from the rhesus draft genome.

### Misassemblies

#### Case 5

serpin peptidase inhibitor, clade B (ovalbumin), member 6 (SERPINB6):

gene split between two chromosomes

**Figure 5:**
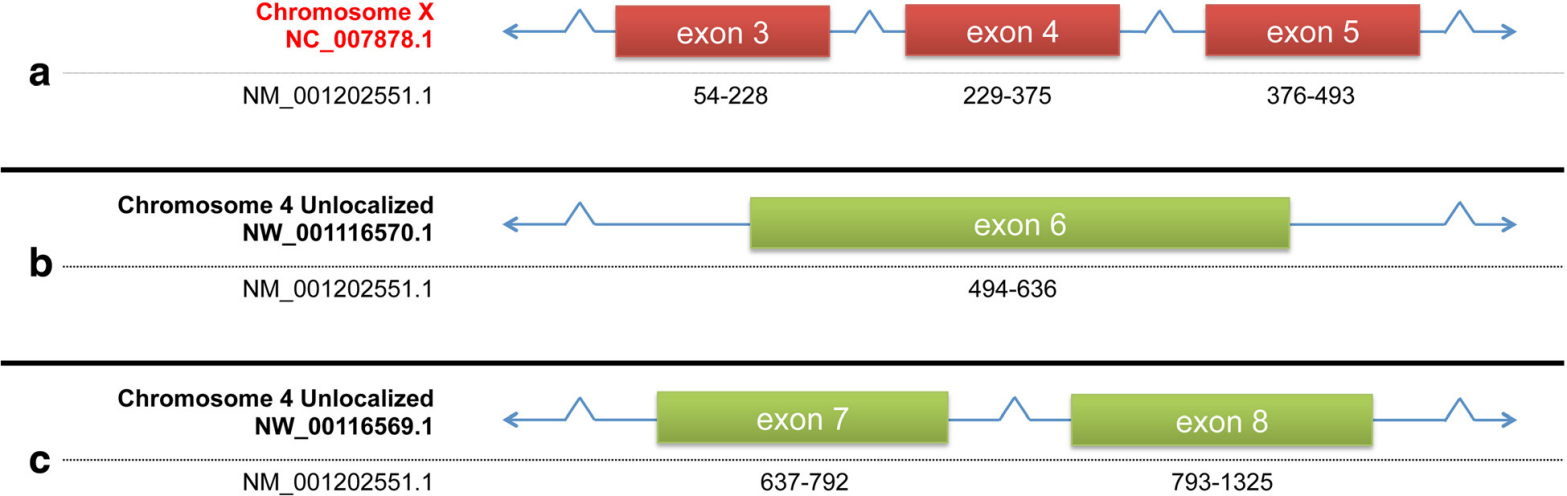
**SERPINB6. Gene split between two chromosomes.** Red exon boxes indicate exons assigned to the wrong chromosome. Green exon boxes indicate exons assigned to the correct chromosome. Accession numbers at left indicate genomic (top) and mRNA (bottom) sequences. Range of mRNA corresponding to exons is indicated by black numbers under exon boxes. Exons not drawn to scale

This apparent misassembly was discovered while trying to build a gene model for the rhesus ortholog of SERPINB6 (Figure 5). The GenBank reference sequence for the mRNA from this gene in rhesus macaques, [GenBank:NM_001202551.1], was built from expressed sequence tags [GenBank:CO583313, CO583313]. Exons 3–5 of this gene in human transcript [GenBank:NM_004568.5] have been assigned to rhesus chromosome X [GenBank:NC_007878.1; position 42,763,676 - 42,768,439]. This genomic sequence, including introns, aligns with human chromosome 6 in the region containing SERPINB6. Exon 6 was assigned to an unlocalized scaffold from rhesus chromosome 4 [GenBank:NW_001116570.1; positions 970–1112] and exons 7 and 8 to another unlocalized scaffold for rhesus chromosome 4 [GenBank:NW_00116569.1; positions 2910–3818]. The rhesus macaque chromosome 4 is syntenic with human chromosome 6, the location of the SERPINB6 gene. Very few genes on a human autosome would be expected to be found on rhesus chromosome X. Therefore, the true location of the rhesus SERPINB6 gene is likely chromosome 4. Contigs which are apparently incorrectly assigned to chromosome X include: [GenBank:AANU01001442.1 and AANU01001441.1]. The correct assignment of these contigs would appear to have been, instead, rhesus chromosome 4.

#### Case 6

RALY RNA binding protein-like (RALYL):

gene split between two chromosomes

**Figure 6:**
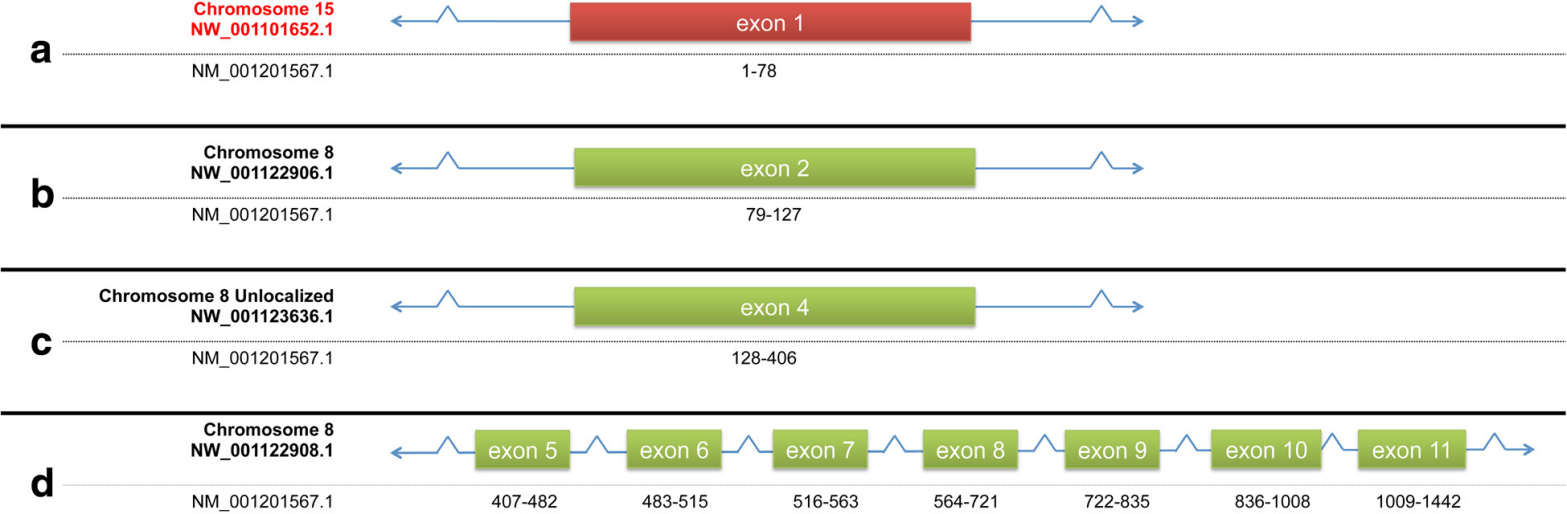
**RALY. Gene split between two chromosomes.** Red exon box indicates exon assigned to the wrong chromosome. Green exon boxes indicate exons assigned to the correct chromosome. Accession numbers at left indicate genomic (top) and mRNA (bottom) sequences. Range of mRNA corresponding to exons is indicated by black numbers under exon boxes. Exons not drawn to scale

This apparent misassembly was discovered while trying to build a gene model for the rhesus ortholog of RALYL (Figure 6). The GenBank reference sequence for the mRNA from this gene in rhesus macaques, [GenBank:NM_001201567.1], contains 125 nucleotides not reported in any other species. However, the CDS begins at position 151. The protein for which this sequence codes is 100% identical to the human ortholog. The first exon of the human RALYL transcript [GenBank: NM_173848.5] appears to be contained in rhesus genomic scaffold [GenBank:NW_001101652.1] (chromosome 15; positions 1–24,914). The second exon is found in the [GenBank:NW_0011236.1] (chromosome 8 unlocalized) sequence. The remainder of the exons are found in [GenBank:NW_001122908.1] (chromosome 8) sequence. Rhesus chromosome 8 is syntenic to human chromosome 8. The contig [GenBank:AANU01164795.1] was apparently incorrectly assigned to chromosome 15. The correct assignment of this contig would appear to have been, instead, rhesus chromosome 8. The anomalous sequence at the 5' end of the rhesus RALY transcript is likely due to this incorrect assignment.

#### Case 7

coiled-coil domain-containing protein 135 (CCDC135):

gene split between two chromosomes

**Figure 7:**
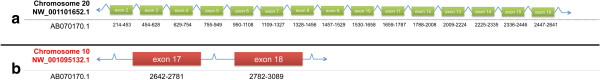
**CCDC135. Gene split between two chromosomes.** Red exon boxes indicate exons assigned to the wrong chromosome. Green exon boxes indicate exons assigned to the correct chromosome. Accession numbers at left indicate genomic (top) and mRNA (bottom) sequences. Range of mRNA corresponding to exons is indicated by black numbers under exon boxes. Exons not drawn to scale

This apparent misassembly was discovered while trying to build a gene model for the rhesus ortholog of CCDC135 (Figure 7). No rhesus ortholog for CCDC135 has been annotated. When the human CCDC135 RNA [GenBank:NM_032269.5] is aligned with the draft rhesus assembly, exons 2–16 map to a genomic scaffold on chromosome 20] while exons 17 and 18 map to a genomic scaffold on chromosome 10 [GenBank:NW_001095132.1]. The same chromosome split is seen when the *Macaca fascicularis* ortholog to CCDC135 [GenBank:AB070170.1] is aligned to the rhesus draft genome sequence. CCDCC13 is found on chromosome 16 in humans. Rhesus chromosome 20 is syntenic with human chromosome 16. Hence, it is likely that the sequence in the contig containing exons 17 and 18 [GenBank:AANU01258958.1] was incorrectly assigned to rhesus chromosome 10. It should have, instead, been assigned to rhesus chromosome 20. [GenBank:AANU01258958.1] appears to be chimeric. The approximately first 8.4 kb of this sequence aligns with human chromosome 20 (which is syntenic with rhesus chromosome 16) while the remainder of this contig aligns with human chromosome 16.

#### Case 8

vacuolar protein sorting 13 homolog D (S. cerevisiae) (VPS13D): gene split between two chromosomes and failure to integrate an unlocalized contig

**Figure 8:**
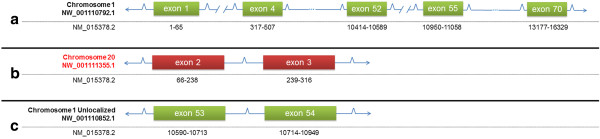
**VPS13D. Gene split between two chromosomes and failure to integrate an unlocalized contig.** Red exon boxes indicate exons assigned to the wrong chromosome. Green exon boxes indicate exons assigned to the correct chromosome. Accession numbers at left indicate genomic (top) and mRNA (bottom) sequences. Range of mRNA corresponding to exons is indicated by black numbers under exon boxes. Forward slashes in panel a indicate breakpoints where genomic fragments were not incorporated in the chromsome 1 file. Three dots were used in panel a to indicate exons not shown (exons 5–51 and 56–69). Exons not drawn to scale

This apparent misassembly was discovered while trying to build a gene model for the rhesus ortholog of VPS13D (Figure 8). This gene is correctly assigned to chromosome 1 by NCBI. However, the rhesus GenBank transcript [GenBank:XM_002802187.1] and protein [GenBank:XP_002802233.1] models for this gene appear to be incorrect. When a blastn [11] alignment is attempted with [GenBank:XM_002802187.1] (default parameters, nr database), no match with any other species for the first 771 nucleotides is found. This span includes exons 1–6 and the 5' region of exon 7. In addition, there is an area of non-alignment at positions 11,045-11,185 of the rhesus model transcript with positions 10,589-10,945 (exons 53 and 54) of the human transcript [GenBank:NM_015378.2]. The anomalous transcript sequences include CDS which explains why attempts to align the rhesus model protein with other species reveal equivalent anomalies (no alignment for amino acids 1–247 and 3683–3730 of the rhesus protein with its human ortholog - [GenBank:NP_056193.2]). Most of the human transcript for VPS13D aligns with the rhesus chromosome 1 [GenBank:NW_001110792.1]. However, regions of this transcript corresponding to exons 2 and 3 align instead with contigs [GenBank:AANU01177793.1, AANU01177791.1] within a scaffold [GenBank:NW_001111355.1] assigned to rhesus chromosome 20. [GenBank:AANU01177793.1] appears to be chimeric. Approximately the first 7,500 nucleotides maps to human chromosome 1. However, the last 4,000 nucleotides map to human chromosome 16, which is syntenic with rhesus chromosome 20. Regions of the human transcript corresponding to exons 53 and 54 were assigned to a rhesus chromosome 1 unlocalized genomic scaffold [GenBank:NW_001110852.1]. This scaffold is derived from a single contig [GenBank:AANU01131929.1]. Thus, it would appear that the incorrect assignment of sequences to chromosome 20 and the failure to integrate a contig into the chromosome 1 file led to two separate inventions of spurious protein sequence by Gnomon.

#### Case 9

Src homology 2 domain containing E (SHE): gene split between two chromosomes

**Figure 9:**
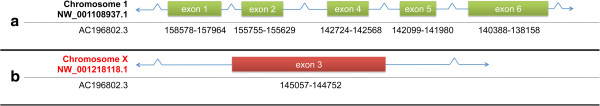
**SHE. Gene split between two chromosomes.** Red exon box indicates exon assigned to the wrong chromosome. Green exon boxes indicate exons assigned to the correct chromosome. Accession numbers at left indicate genomic (top) and mRNA (bottom) sequences. Range of mRNA corresponding to exons is indicated by black numbers under exon boxes. Exons not drawn to scale

This apparent misassembly was discovered while trying to build a gene model for the rhesus ortholog of SHE (Figure 9). The rhesus ortholog of SHE is correctly assigned to chromosome 1. However, it has a provisional gene symbol LOC716722 [9] and the gene description: SH2 domain-containing adapter protein E-like. The "like" may have been added to this gene description because attempts to align the transcript and protein, [GenBank:XM_002801807.1 and XP_002801853.1], respectively, with the human transcript [GenBank:NM_001010846.2] and protein [GenBank:NP_001010846.1] fail in the region corresponding to exon 3. Specifically, the proposed rhesus protein appears to lack amino acids 240–339. This is apparently because part of the contig containing rhesus SHE exon 3 [GenBank:AANU01113464.1] was mistakenly assigned to chromosome X [GenBank:NW_001218118.1]. Further evidence for the misassembly of the draft rhesus genome can be found in the sequence of a rhesus BAC clone [GenBank:AC196802.3] which contains the entire rhesus ortholog of SHE, including exon 3, and maps to rhesus chromosome 1. Contig [GenBank:AANU01113464.1] appears to be chimeric as it contains some sequence belonging to rhesus chromosome 1 (approximately 1–2,000) and some sequence belonging to rhesus chromosome X (approximately 2,000 - 5,282).

#### Case 10

Bardet-Biedl syndrome 1 (BBS1):

genomic fragment containing exon in wrong orientation

**Figure 10:**
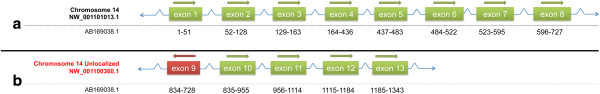
**BBS1. Genomic fragment containing exon in the wrong orientation.** Red exon box indicates exon in the wrong orientation with respect to the other exons in this gene. Green exon boxes indicate exons in the correct orientation. Accession numbers at left indicate genomic (top) and mRNA (bottom) sequences. Range of mRNA corresponding to exons is indicated by black numbers under exon boxes. Exons not drawn to scale

This apparent misassembly was discovered while trying to build a gene model for the rhesus ortholog of BBS1 (Figure 10). No rhesus ortholog for BBS1 has been annotated in GenBank. This is because the contig [GenBank:AANU01213737.1] containing exon 9 of the rhesus ortholog of the human BBS1 gene [GenBank:NM_024649.4] is in a different orientation with respect to other exons of this gene in the same genomic scaffold [GenBank:NW_001100360.1]. A cynomolgus macaque transcript which is an ortholog to the human BBS1 gene has been reported - [GenBank:AB169038.1]. The existence of this transcript in a closely related species to the rhesus macaque provides further evidence indicating that the rhesus draft assembly likely contains a contig in the wrong orientation in this region.

### Chromosome 20 misannotations

**Figure 11:**
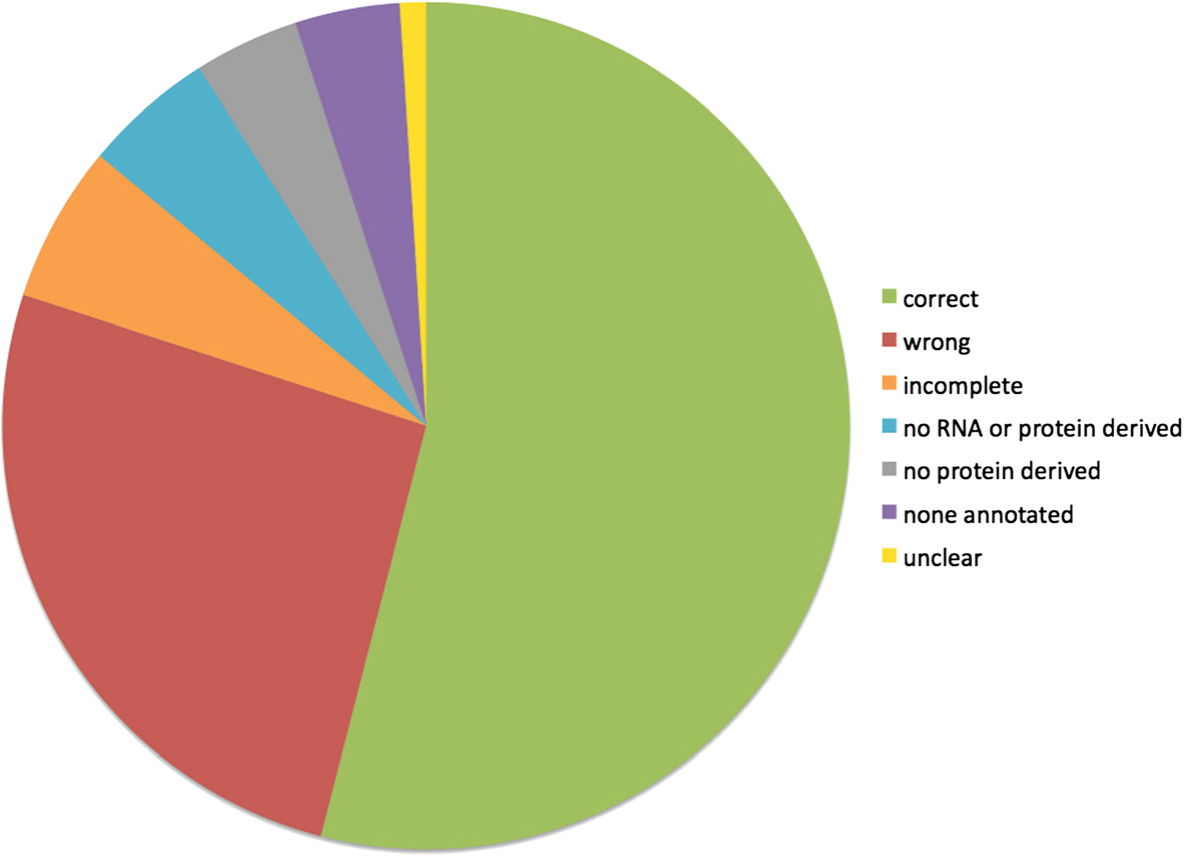
Pie chart illustrating categories of gene annotations

To estimate the frequency of misannotations of rhesus genes, the first 100 genes from rhesus chromosome 20 were examined for evidence of misannotation (Additional file 1: Supplementary Table 1). Because rhesus chromosome 20 is syntenic with human chromosome 16, it is possible to directly compare human genes with their putative rhesus orthologs. Only 54% of the proteins associated with this set appeared to be complete and correct (Figure 11, Additional file 1: Supplementary Table 1). 26% appeared to be wrong, 6% incomplete, 5% no RNA or protein derived, 4% none annotated and 1 was unclear.

## Discussion

The draft rhesus assembly was described in a publication which appeared in April 2007 [1]. As with all draft sequences, there were substantial gaps in the assembly as well as sequencing errors. How significant were these errors? Global statistics were provided in the paper describing this assembly [1]. However, these types of statistics, while providing useful general information, can leave the reader with an incorrect perception of completeness at the gene level. For example, although it is reported that "98% of the available genome was represented" [1], we estimate that approximately 50% of the gene models are incomplete or incorrect leading to missing, incomplete or spurious proteins. This was partly due to sequencing errors. A single missing nucleotide in an exon can result in a seriously incorrect protein model. Although the mistake represents a very small percent of the total draft sequence, the consequences to investigators relying on the accuracy of the gene models can be very great.

Misannotation of genes, transcripts and proteins with Gnomon and other automated pipelines has been previously reported [12,13]. Further, a recent study using exome data found significant errors in sequence and annotation in rhesus macaques and chimpanzees [14]. According to the documentation for Gnomon [9], empirical evidence is preferred when producing gene models. However, for many draft species (including the rhesus macaque), complete transcriptome information is lacking. In the absence of such information, Gnomon attempts to predict exons from genomic sequence [9]. If this sequence is incomplete or wrong, there is no indication that Gnomon is able to detect or even flag this possibility. Indeed, we observed instances where Gnomon “invented” exons from intronic sequence when a complete exon was not present. As a result, both transcript and protein models were incorrect. We also found instances of genes incorrectly annotated as pseudogenes because Gnomon was unable to build a complete gene model due to missing or incorrect sequence information. It is important to point out that the errors made by Gnomon with the rhesus macaque genome assembly would likely be made with any automated gene model pipeline with any draft vertebrate genome. Hence, the issues identified here represent a pervasive challenge to the analysis of all draft vertebrate genomes.

In the paper describing the draft rhesus genome, 1.8 Mb of finished sequence was compared to the available ESTs to determine whether there were misassemblies [1]. It was reported that: "No misassemblies were identified in that comparison" [1]. However, our gene-based approach indicates that it is highly likely that there were a number of significant misassemblies, including contigs assigned to the wrong chromosomes. The discovery that some of the contigs were chimeric brings into question the assembly of the contigs themselves. The total number of misassemblies will not be known until a more finished version of the rhesus macaque genome is available for comparison. However, there are two studies which also support our contention that there are major misassemblies in the rhesus genome [15,16].

Draft sequences can be very helpful for some tasks. For example, we used a preliminary draft version of the rhesus macaque genome in our targeted approach to rhesus macaque microarray design [17]. However, for other uses, draft genomes may be insufficient to fully utilize genomics approaches, or worse, result in the generation of spurious results. Most investigators prefer to align their NextGen sequences against a reference genome. This cannot be reliably done with the rhesus draft genome (and likely not with other draft vertebrate genomes). Other methods which require a high quality reference genome include: SNP discovery and nucleotide-based probes (exon capture, siRNA and Quantitative PCR).

Modern evolutionary studies depend on alignments of genomic sequences between species. Mismatches between species can be interpreted as indications of evolutionary change. However, given the incidence of sequencing errors and misannotation in the rhesus macaque draft genome (and likely all vertebrate draft genomes), such assumptions may lead to incorrect results. The more draft genomes included in an analysis, the smaller the subset of genes correctly annotated across all the genomes included in the analysis. This is a particular challenge to evolutionary studies which require information from multiple species.

Our exon order-based approach to identifying misassemblies suggests a possible strategy for assessing the quality of genome assemblies. Since exon order is highly conserved among vertebrates, requiring that contigs containing exons respect this order should lead to improved assemblies.

## Conclusions

To take full advantage of the rhesus macaque genome, or indeed, any vertebrate genome, standard draft coverage (5-6X Sanger sequencing) is insufficient. This is because small sequence errors or gaps can result in large errors in assembly and in annotation. Given the relative costs involved, it may make sense to bring draft genomes closer to finished quality using NextGen sequencing.

## Methods

### Reference sequence and assembly

The initial draft rhesus assembly Mmul_0.1 (rheMac1) was released in January 2005. Mmul_051212 (rheMac2) was released in January 2006. No further assemblies based on this animal have been released since Mmul_051212. The draft rhesus genome assembly discussed in this work refers to Mmul_051212.

NCBI produced a new annotation of Mmul_051212 in April 2010 (*Macaca mulatta* build 1.2) using Gnomon, a gene prediction tool for eukaryotes [9]. The annotations discussed in this work refer to build 1.2 annotations. The NCBI database Gene [10] was used to determine annotations for the rhesus draft genome.

### Targeted re-sequencing

To assess the types of sequencing errors which occurred in the rhesus draft genome, 4 suspect exons from different genes were identified by attempting to align human mRNA with the rhesus draft genome. Evidence of likely error in annotation consisted of amino acids in the rhesus model not found in other species or a truncated protein with respect to other species. PCR primers were designed which flanked these exons using Primer3 [18,19]. Default settings were used with the following exception: the human mispriming library was selected. Rhesus genomic DNA obtained from the reference animal was used as the template for amplification using standard PCR procedures [20]. Sanger sequences were obtained and used to determine whether the draft rhesus genome sequences were correct. All sequences were deposited in GenBank [HM067826.1, JF749838.1, JN589014.1, JN624744.1]. Four cases are described in detail in the Results section (Cases 1–4).

### Misassembly detection

Vertebrate exon order is highly conserved. We took advantage of this fact to identify potential misassemblies in the rhesus macaque draft genome. BLAST was used to align human mRNAs against the draft rhesus chromosome and scaffold files. Anomalies in exon order were noted. Additional evidence for misassemblies consisted of mRNA data from rhesus macaques or a closely related species - the cynomolgus macaque (*Macaca fascicularis*) and sequence from a BAC clone. Six cases are described in detail in the Results section (Cases 5–10).

### Estimation of frequency of misannotations

The first 100 genes of rhesus chromosome 20 were compared with the orthologous human genes using the same rules which identified the four genes targeted for Sanger sequencing. Human chromosome 16 is syntenic with rhesus chromosome 20. Both similarity and synteny information were used to identify misannotations.

## Competing interests

The author(s) declare that they have no competing interests.

## Authors' contributions

RN conceived the study, analyzed sequence and misannotation errors and drafted the manuscript. XZ identified some of the misassemblies, contributed to analysis of the first 100 rhesus genes on chromosome 20 and helped draft the manuscript. JG analyzed the sequences, performed the PCRs and contributed to the draft of the manuscript. All authors read and approved the final manuscript.

## Supplementary Material

Additional file 1 Supplementary Table 1. List of misannotations of the first 100 genes on rhesus chromosome 20. Click here for file
